# Loss of MeCP2 Function Across Several Neuronal Populations Impairs Breathing Response to Acute Hypoxia

**DOI:** 10.3389/fneur.2020.593554

**Published:** 2020-10-30

**Authors:** Christopher S. Ward, Teng-Wei Huang, Jose A. Herrera, Rodney C. Samaco, Christopher M. McGraw, Diana E. Parra, E. Melissa Arvide, Aya Ito-Ishida, Xiangling Meng, Kerstin Ure, Huda Y. Zoghbi, Jeffrey L. Neul

**Affiliations:** ^1^Department of Molecular Physiology and Biophysics, Baylor College of Medicine, Houston, TX, United States; ^2^Jan and Dan Duncan Neurological Research Institute, Texas Children's Hospital, Houston, TX, United States; ^3^Department of Molecular and Human Genetics, Baylor College of Medicine, Houston, TX, United States; ^4^Program in Developmental Biology, Baylor College of Medicine, Houston, TX, United States; ^5^Interdepartmental Program in Translational Biology and Molecular Medicine, Baylor College of Medicine, Houston, TX, United States; ^6^Department of Pediatrics, Baylor College of Medicine, Houston, TX, United States; ^7^Department of Neuroscience, Baylor College of Medicine, Houston, TX, United States; ^8^Howard Hughes Medical Institute, Baylor College of Medicine, Houston, TX, United States

**Keywords:** Rett, MeCP2, hypoxia, sudden death, biomarker, breathing abnormalities, genetic manipulation, pharmacological manipulation

## Abstract

Rett Syndrome (RTT) is a neurodevelopmental disorder caused by loss of function of the transcriptional regulator Methyl-CpG-Binding Protein 2 (MeCP2). In addition to the characteristic loss of hand function and spoken language after the first year of life, people with RTT also have a variety of physiological and autonomic abnormalities including disrupted breathing rhythms characterized by bouts of hyperventilation and an increased frequency of apnea. These breathing abnormalities, that likely involve alterations in both the circuitry underlying respiratory pace making and those underlying breathing response to environmental stimuli, may underlie the sudden unexpected death seen in a significant fraction of people with RTT. In fact, mice lacking MeCP2 function exhibit abnormal breathing rate response to acute hypoxia and maintain a persistently elevated breathing rate rather than showing typical hypoxic ventilatory decline that can be observed among their wild-type littermates. Using genetic and pharmacological tools to better understand the course of this abnormal hypoxic breathing rate response and the neurons driving it, we learned that the abnormal hypoxic breathing response is acquired as the animals mature, and that MeCP2 function is required within excitatory, inhibitory, and modulatory populations for a normal hypoxic breathing rate response. Furthermore, mice lacking MeCP2 exhibit decreased hypoxia-induced neuronal activity within the nucleus tractus solitarius of the dorsal medulla. Overall, these data provide insight into the neurons driving the circuit dysfunction that leads to breathing abnormalities upon loss of MeCP2. The discovery that combined dysfunction across multiple neuronal populations contributes to breathing dysfunction may provide insight into sudden unexpected death in RTT.

## Introduction

Rett syndrome (RTT, OMIM 312750) is a neurodevelopment disorder caused by mutations in the X-linked gene *Methyl-CpG Binding Protein 2* (*MECP2*) that is characterized by initial normal development followed by regression manifesting as loss of acquired skills (1–3). Many affected individuals also develop autonomic and physiological deficits including disrupted breathing with bouts of hyperventilation and apnea (4, 5). Approximately one quarter of all deaths in RTT are sudden and unexpected, raising the possibility that breathing abnormalities contribute to some of these deaths (6). The abnormal breathing pattern may indicate deficits in the networks that maintain normal respiratory rhythmogenesis as well as those that modify its response to environmental stimuli (7).

Several mouse models have been generated that display features similar to the human disorder (8–11). While female mice heterozygous for *Mecp2* mutations show many features reminiscent of the human disorder, the nature of the X-linked mutation also causes them to be mosaic for MeCP2 and less severely affected than hemizygous male animals. For these reasons, male mice are often used to help dissect anatomic and neuronal population effects of MeCP2 expression. Mice lacking MeCP2 function exhibit an abnormal response to hypoxia (12–14). Normally during acute hypoxia blood oxygen status is sensed by the carotid body located along the carotid bifurcation and relayed to brainstem respiratory centers through the nucleus tractus solitarius (NTS) (15–17). The expected response to an acute hypoxic challenge in adult mammals is an immediate increase in ventilation in an effort to increase gas exchange and blood oxygenation, within a timescale of minutes, ventilation then decreases as the failure to improve blood oxygen saturation persists and conservation of the remaining oxygen for essential body functions becomes critical (16–19). The increase in ventilation followed by ventilatory decline until ventilation approaches baseline levels is commonly referred to as the hypoxic ventilatory response, with the ventilatory decline stage often referred to as hypoxic ventilatory decline (HVD) (16, 20, 21).

However, in animals lacking Mecp2, the breathing rate during acute hypoxia remains elevated relative to that of wildtype littermates (12, 14, 22). In combination with disordered breathing characterized by bouts of hyperventilation interspersed with apnea, the persistent increase in ventilation in a hypoxic environment may be maladaptive and creates risk from increased metabolic demand without sufficient supply of oxygen (23). Furthermore, the maladaptive reflexive breathing response to external stimuli observed in these animals may reflect the underlying circuit-level disruption that might contribute ultimately to sudden unexpected death. A key unanswered question is the neuronal cell-type contribution to the abnormal hypoxia response in MeCP2 mutant animals. To investigate the underlying etiology of the abnormal HVD that occurs in mice lacking MeCP2, we used a combination of genetic and pharmacological methods to dissect the cellular contribution to the HVD. We identified that mice lacking MeCP2 acquire a deficit in HVD, presenting with a persistently increased breathing rate during exposure to hypoxia. Furthermore, we identified that MeCP2 function within excitatory, inhibitory, and modulatory neuronal populations is critical for a normal HVD.

## Materials and Methods

### Animals Used in Experiments

All research and animal care procedures were approved by the Baylor College of Medicine Institutional Animal Care and Use Committee and housed in the Association for Assessment and Accreditation of Laboratory Animal Care-approved animal facility at Baylor College of Medicine. *Mecp2*^*Tm*1*Bird*^ mice were obtained as a gift from Dr. Adrian Bird (University of Edinburgh, Edinburgh, UK, RRID:IMSR_JAX_007177) and backcrossed and maintained on a 129S6 background for >10 generations (8). *Mecp2*^*Tm*1.1*Bird*^ mice were generated via Cre mediated recombination of *Mecp2*^*Tm*1*Bird*^ mice and maintained on a 129S6 background. *Mecp2*^*Tm*2*Bird*^ mice were obtained from The Jackson Laboratory and maintained on a C57BL/6J background (RRID:IMSR_JAX_006849) (9). *Mecp2*^*TM*1.1*Bird*^ mice used for experiments were generated by crossing the congenic 129S6 *Mecp2*^*TM*1.1*Bird*/+^ female mice to wild-type male C57BL/6J mice to generate male 129B6F1 *Mecp2*^+/*Y*^ (WT) and *Mecp2*^*Tm*1.1*Bird*/*Y*^ (NULL) mice, and female *Mecp2*^+/+^ (WT) and *Mecp2*^*Tm*1.1*Bird*/+^ (HET) mice. *Nestin-Cre* (RRID:IMSR_JAX_003771) mice were maintained on a C57BL/6J background; *Th-Cre* (MGI:2677450), and *VIAAT-Cre* (MGI:4867735) and *VGLUT2-Cre* (RRID:IMSR_JAX_016963) mice were maintained on a FVB/NJ strain background. Cre lines were crossed to *Mecp2*^*Tm*1*Bird*^, or *Mecp2*^*Tm*2*Bird*^ to generate male conditional knockout and conditional rescue mice and strain matched control littermates (24–27).

### Unrestrained Whole Body Plethysmography

Unrestrained plethysmography in mice was performed similarly to methods previously described (14, 22). Mice were placed within unrestrained whole-body plethysmography chambers (Buxco), ~500 mL in volume with a continuous flow rate of 500 mL/min flushing the chambers with fresh air. Mice were allowed to acclimate for at least 20 min, and baseline breathing was then recorded for 30 min. Data were collected during each animals' session within the plethysmograph chambers without additional pre-data collection habituation sessions. To determine response to hypoxic gas (10% O_2_, balance N_2_), the chamber was then flushed with hypoxic gas for 20 min. Breathing rate was determined using a customized algorithm written in Matlab (RRID:SCR_001622) using the plethysmography signal files captured with Ponemah3 software (RRID:SCR_017107). To reduce the artifacts from excessive movement and sniffing behavior, breaths that exhibited an inspiratory time <0.03 s, an expiratory time >10 s, and a calculated exhaled tidal volume >150 or <50% of calculated inhaled tidal volume were excluded; breaths were then filtered with intervals excluded if they possessed >10% of their breaths above 500 breaths per minute (sliding 200 breath windows centered on the current breath). The peak breathing rate during hypoxia was calculated as the 90th percentile for breaths during the first 5 min of hypoxic challenge. Breathing parameters for each animal during baseline and as well as during the declined phase of hypoxic challenge (at 10 min from the onset of hypoxia until 15 min) were determined as the average instantaneous value over the recorded interval and then averaged across trials for animals subjected to repeated measurements. Additional parameters were collected during the baseline portion and include apnea index (apneas per 10,000 breaths, defined as a breath >0.5 s in duration and > 2x the overall baseline breath duration and >2x the duration of the 6 surrounding breaths), irregularity score calculated from breath duration changes (|[n]-[n_−1_]|/[n_−1_]), and uncompensated tidal volume calculated from the box flow of air into and out of the plethysmograph chamber. Respiratory parameters were then compared across genotypes, sex, or treatment as described in the text.

### Pharmacological Manipulation of Hypoxic Breathing Response

Adult male 129B6F1 WT and NULL mice were treated with sterile phosphate buffered saline or drugs dissolved in sterile phosphate buffered saline by intraperitoneal injection immediately preceding plethysmography recordings of the hypoxic breathing response as described above. Following a minimum 48 h “wash-out” period, mice were tested with another drug and dosage combination. Drug and doses used include tiagabine at 3, 10, and 30 mg/kg; muscimol at 1, 2, and 3 mg/kg; baclofen at 1, 2, and 3 mg/kg; desipramine at 3, 10, and 30 mg/kg; ketamine at 10 and 30 mg/kg; and L-DOPA at 30, 60, or 100 mg/kg administered alone or with carbidopa at 10 mg/kg.

### Histological Quantification of Neuronal Activity

Quantification of hypoxia induced neuronal activity and regions selected for analysis were adapted from methods used to look at hypoxia induced activity in the rat brain (28). To reduce artifacts of novelty and stress induced neuronal activation, male mice were habituated to the unrestrained whole body plethysmography chambers for at least 1 h a day for 4 days before being submitted to a gas challenge. On the day of the gas challenge 129B6F1 WT and NULL mice were placed in the plethysmography chambers and subjected to room air for 30 min to establish baseline breathing, mice were then treated for 3 h with hypoxic gas (10% O_2_, balance N_2_) or treated with room air to control for basal neuronal activity. Mice were then immediately anesthetized and processed for immuno-histology.

Male mice were deeply anesthetized by intraperitoneal injection with Avertin at a dose of 0.04 ml/g and then fixed by transcardiac perfusion with PBS followed by 4% paraformaldehyde in PBS. Tissues for histological analysis were harvested and fixed overnight in 4% paraformaldehyde in PBS and cryopreserved by overnight incubations in increasing concentrations of sucrose (up to 30% sucrose). Tissues were embedded in O.C.T. compound (Sakura) and sectioned. Sections were collected for at a thickness of 50 μm and stained as floating sections. Sections were blocked for 1 h in a PBS solution containing 10% serum (matched to the host used for the secondary antibodies) and 0.3% Triton X-100. Neuronal activation was assayed by immune-staining for c-Fos expression with a rabbit-anti-c-Fos antibody diluted 1:500 (RRID:AB_2106783), and a goat-anti-rabbit secondary antibody conjugated to dylight 549 (JaxIR). Primary and secondary antibody incubation was performed in the blocking solution for 12–48 h at 4°C or 6 h at room temperature. Sections were washed between incubations with PBS and 0.05% Triton X-100. If not included in the mounting medium, DAPI was included in the penultimate wash. Sections were mounted with Prolong mounting medium (Invitrogen) and imaged via epifluorescent microscopy (Zeiss M1 with Axiovision software, RRID:SCR_002677).

Quantification of neuronal activation bilaterally across regions in the hindbrain was performed via stereological estimation with an optical dissector excluding the upper surface of the tissue, a slide sampling fraction of 1/3 and an area fractionator of 1/4. Image processing and counting of neurons was performed using ImageJ software (29). NTS, locus coeruleus (LC), and Parabrachial Complex (PB) regions were identified based on their anatomic locations as defined in the Paxinos mouse brain atlas (30). Quantified neurons are expressed as the number of neurons bilaterally present in the region per mouse.

### Statistics

All statistics were performed using SPSS (RRID:SCR_002865) on a PC. Parametric statistics were performed using ANOVA for the indicated factors. For conditions where factor had more than two levels, formalized *post-hoc* testing was performed using Student-Newman-Keuls or Bonferroni correction for multiple comparisons to detect pairwise differences.

## Results

### Loss of MeCP2 Function Impairs the Breathing Rate Response to Hypoxia

**Figure 1:**
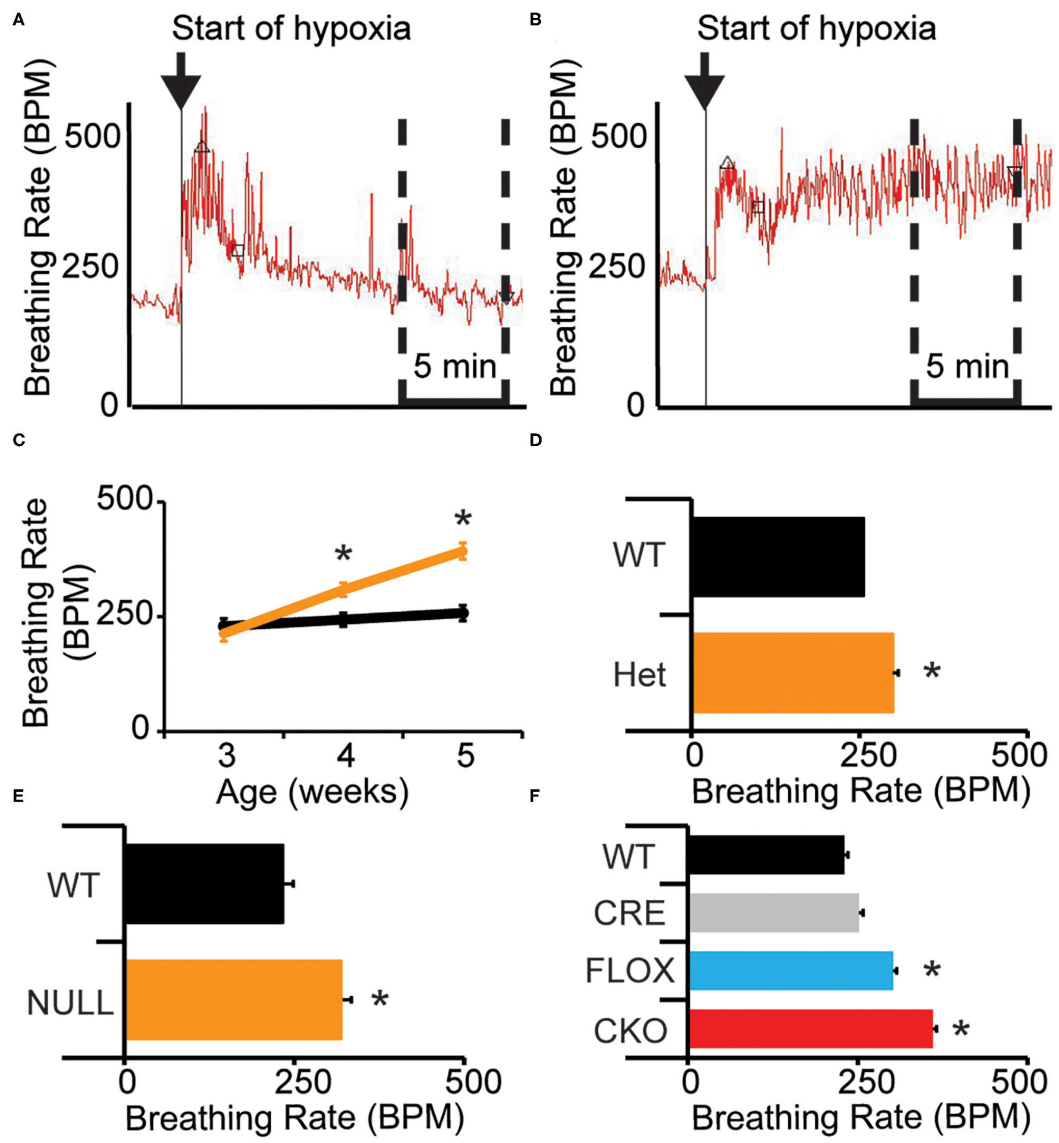
Loss of MeCP2 function disrupts the breathing rate response to acute hypoxia. **(A)** Wild-type mice exhibit a typical breathing rate response to exposure to hypoxia, with an immediate increase in breathing rate followed by a decline to near baseline breathing rates—representative image shown. Upward pointed triangle indicates timing of peak hypoxic breathing, defined as the 90th percentile for breathing rate during the first 5 min of hypoxic challenge, square indicates the third minute of the hypoxic challenge. **(B)** NULL mice exhibit an abnormal breathing rate response to hypoxia, with an immediate and persistent increase in breathing rate for the duration of exposure to hypoxia—representative image shown. The time segment used to determine the average “declined breathing rate” between 10 and 15 min after the onset of hypoxia is shown by the dashed box in **(A,B)**. The “declined breathing rate” is presented in **(C–F)**. **(C)** Male 129B6F1 NULL mice acquire the elevated “declined breathing rate” after 3 weeks of age **(D)**. This elevated “declined breathing rate” is also present in aged female HET mice, **(E)** and in male NULL mice on a C57BL/6J background. **(F)** Removal of MeCP2 function from the nervous system with *Nestin-Cre* also causes the elevated breathing rate during hypoxia. **(C–F)** indicate mean +/– S.E.M. *p < 0.05 effect of genotype by ANOVA with Student-Newman-Keuls *post-hoc* correction for multiple comparisons for **(F)** (CKO vs. all other genotypes). Number of animals in each group are indicated in Tables 1, 2. Representative plethysmography traces are available (Supplementary Figures 1, 2).

Mice lacking MeCP2 (male-NULL and female-HET) exhibit a higher breathing rate during exposure to hypoxia (10% O2, balance N2) than their wild-type littermates (14). We sought to better understand the nature of this abnormal breathing response using unrestrained whole body plethysmography. On a 129B6F1 isogenic strain background, we observed that similar to WT mice, NULL mice show a sharp increase in their breathing rate in response to acute hypoxia; however, NULL mice maintain a persistently elevated breathing rate as opposed to WT mice that exhibit HVD and decrease their breathing rate to near baseline levels as the exposure to hypoxia continues within the first 5 min after initiation of hypoxia (Figures 1A,B, Table 1).

**Table 1:** Baseline and hypoxic breathing parameters in *Mecp2* mutant mice grouped by age, strain, and sex.

**Strain**	**Sex**	**Age**	**Genotype**	***N***	**Baseline BPM^**∧**^**	**10% O2 Peak BPM**	**10% O2 Declined BPM+^**∧**^**
129B6F1	Male	3 wk	WT	13	255.54 ± 32.81	479.08 ± 98.78	229.14 ± 41.36
			NULL	12	243.19 ± 25.73	460.82 ± 45.18	213.75 ± 74.83
		4 wk	WT	8	240.74 ± 12.71	494.00 ± 61.13	243.96 ± 24.03
			NULL	8	271.32 ± 20.82*	447.55 ± 33.48	308.58 ± 53.47*
		5 wk	WT	7	237.05 ± 10.10	504.73 ± 59.67	258.04 ± 39.14
			NULL	6	260.22 ± 18.58*	470.79 ± 36.29	399.10 ± 36.11*
129B6F1	Female	2 yr	WT	7	240.39 ± 17.65	484.56 ± 46.72	255.87 ± 28.50
			HET	8	296.92 ± 38.55*	430.07 ± 35.58*	299.59 ± 16.36*
C57BL/6J	Male	2 mo	WT	10	279.24 ± 68.88	585.32 ± 32.56	233.01 ± 22.71
			NULL	11	287.00 ± 32.96	516.48 ± 91.96*	318.92 ± 77.95*

Mean breath per minute (BPM) values are presented, +/– SD. *p < 0.05 effect of genotype (within each age, strain, and sex group). +p < 0.05 effect of age (Male 129B6F1 only). ^∧^p < 0.05 effect of age*genotype (Male 129B6F1 only) as determined by ANOVA. Additional parameters including Irregularity Score, Apnea Index, and uncompensated Tidal Volume were also quantified (Supplementary Table 1). Representative plethysmography traces are available (Supplementary Figure 1).

Prior studies have demonstrated that the onset of apnea and disrupted breathing within this model are acquired during the first few weeks of life, progress across the lifespan of the animal, and can be exacerbated by hypoxic or hypercapnic challenge (31, 32). We sought to determine whether the deficit in HVD also is acquired as the animals age. We quantified HVD by determining the average “declined breathing rate” measured from 10 to 15 min after the onset of hypoxia. We observed normal patterns of HVD in NULL mice relative to control littermates at 3 weeks of age. By 4 weeks of age, NULL mice begin developing a significant HVD deficit that continues to worsen at 5 weeks of age. WT mice show similar patterns of HVD across the tested ages (Figure 1C, Table 1).

We also wanted to examine if this phenotype was robust enough to be observed across sexes and strain backgrounds. On a 129B6F1 strain background, 2 year old female HET mice reproduce the impaired HVD observed in the males (Figure 1D, Table 1). Similarly, male NULL mice raised on a C57BL/6J strain background also have an increased breathing rate during exposure to hypoxia (Figure 1E, Table 1). The trend toward a slight decrease in the peak breathing rate during hypoxic exposure observed in the 129B6F1 male NULL mice, is significant for the female HET mice and C57BL/6J male NULL mice (Table 1).

### MeCP2 Function in Excitatory, Inhibitory, and Modulatory Neurons Critical for the Breathing Rate Response to Hypoxia

To better define in which neuronal cell-types MeCP2 expression is required for a normal HVD, we performed genetic removal and restoration of *Mecp2*. This produced wild-type (WT) mice, mice possessing just the *Cre* alleles (CRE), mice possessing just the *Mecp2*^*Tm*1*Bird*^ allele (FLOX) or double mutant mice possessing both *Cre* and *Mecp2*^*Tm*1*Bird*^ alleles (conditional knock out—CKO). The CKO mice are deficient for MeCP2 activity within the cell populations that expressed the cre-recombinase. For genetic rescue studies, we generated WT and CRE mice as well as mice possessing just the *Mecp2*^*Tm*2*Bird*^ allele (STOP) or both *Cre* and *Mecp2*^*Tm*2*Bird*^ alleles (RESC). The STOP mice do not express MeCP2 due to a Cre excisable STOP cassette within the endogenous *Mecp2* locus, while RESC mice have MeCP2 activity solely restored to cells that have expressed the cre-recombinase.

**Figure 2:**
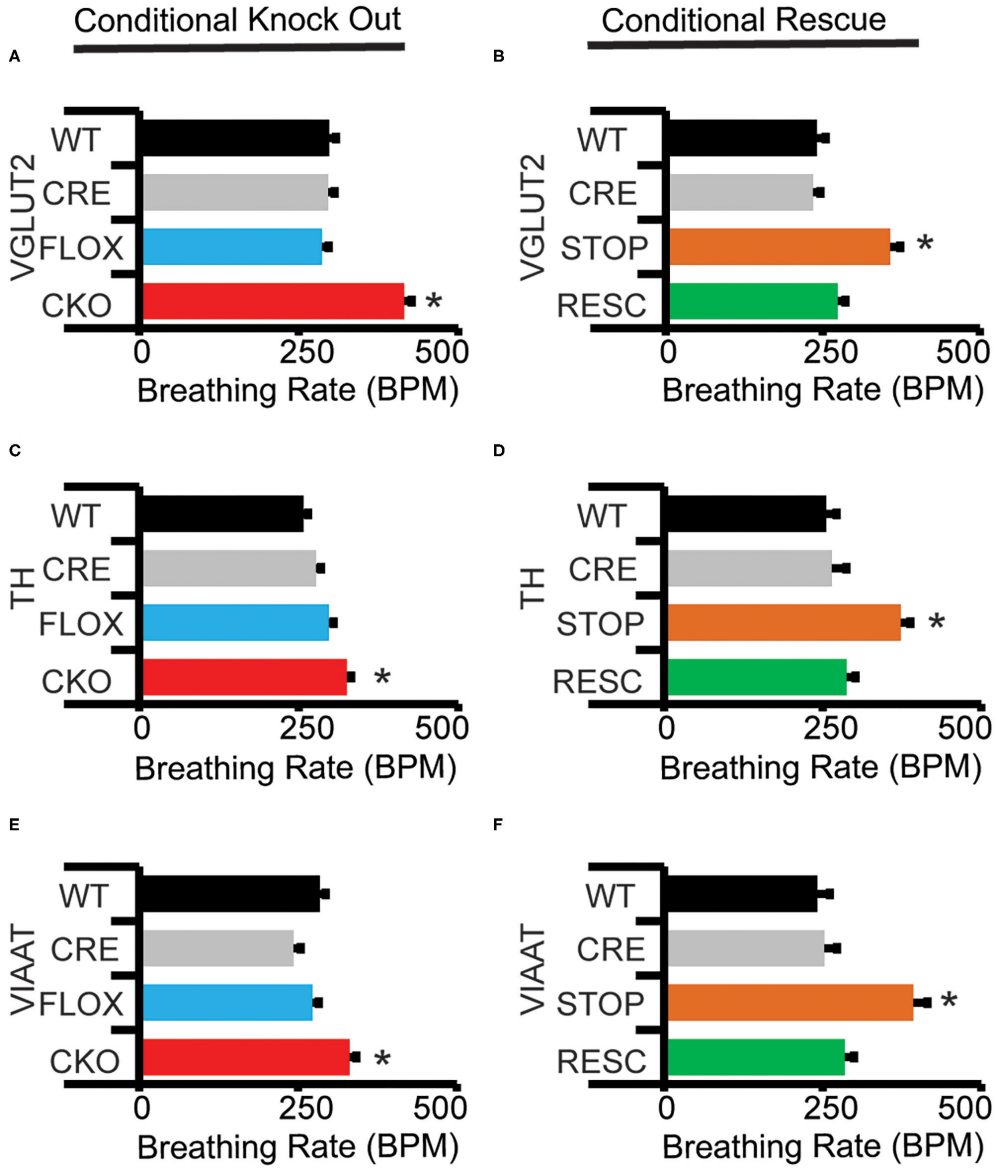
Conditional knock-out and conditional rescue of MeCP2 shows necessity and sufficiency of MeCP2 expression for hypoxic breathing decline. “Declined breathing rate” (between 10 and 15 min after the onset of hypoxia) data is presented for genetic conditional experiments generated with **(A,B)** *VGLUT2-Cre*, **(C,D)** *TH1-Cre*, and **(E,F)** *VIAAT-Cre*. Values indicate mean +/– S.E.M. *p < 0.05 effect of genotype by ANOVA with Student-Newman-Keuls *post-hoc* correction for multiple comparisons (indicated genotype vs. all other genotypes). ns not significant. Number of animals in each group is presented in Table 2. Representative plethysmography traces are available (Supplementary Figure 2).

CKO mice produced with Nestin-Cre, lack MeCP2 function within the nervous system and reproduce the impaired HVD observed in the NULL animals (Figure 1F, Table 2). Additionally, CKO mice with MeCP2 function removed from excitatory (VGLUT2-CKO), inhibitory (VIAAT-CKO) and modulatory neuronal populations (TH-CKO) all show significant impairment in HVD (Figures 2A,C,E, Table 2). Furthermore, RESC mice with MeCP2 function restored to excitatory (VGLUT2-RESC), inhibitory (VIAAT-RESC), and modulatory (TH-RESC) neuronal populations show significant improvement of their HVD relative to their STOP littermates (Figures 2B,D,F, Table 2).

**Table 2:** Baseline and hypoxic breathing parameters from *Mecp2* conditional knockout and conditional rescue mice.

**CKO/CR**	**Cre**	**Genotype**	***N***	**Baseline BPM**	**10% O2 Peak BPM**	**10% O2 Declined BPM**
CKO	Nestin	W	11	217.39 ± 41.73	464.07 ± 79.87	230.23 ± 30.91
		C	9	233.48 ± 35.36	495.70 ± 80.45	251.81 ± 38.92
		F	11	256.67 ± 43.43	517.61 ± 44.85	301.87 ± 55.38*
		K	13	307.59 ± 52.13*	482.24 ± 53.72	360.97 ± 63.10*
	Vglut2	W	13	246.61 ± 47.30	539.80 ± 28.46	297.66 ± 50.85
		C	15	239.39 ± 33.31	524.35 ± 24.36	295.67 ± 51.18
		F	14	264.06 ± 44.39	542.15 ± 26.40	286.42 ± 28.71
		K	11	293.19 ± 30.52	503.16 ± 17.60*	415.00 ± 18.42*
	Th1	W	8	219.34 ± 14.79	499.32 ± 60.94	257.22 ± 39.04
		C	7	223.88 ± 34.53	549.64 ± 43.61	276.71 ± 36.10
		F	8	237.20 ± 24.44	530.36 ± 61.40	297.02 ± 14.70
		K	11	226.38 ± 34.37	538.47 ± 39.43	325.28 ± 15.42*
	Viaat	W	8	264.12 ± 55.73	520.36 ± 47.03	284.30 ± 21.17
		C	5	236.23 ± 38.06	497.89 ± 50.76	242.84 ± 26.54
		F	7	273.85 ± 66.86	503.83 ± 53.57	272.89 ± 40.04
		K	6	240.39 ± 23.17	472.51 ± 44.40	331.26 ± 39.22*
CR	Vglut2	W	6	219.58 ± 22.79	536.62 ± 48.76	240.16 ± 49.81
		C	9	250.67 ± 53.49	531.37 ± 72.05	234.40 ± 31.19
		S	5	299.98 ± 74.80	509.45 ± 61.14	357.19 ± 64.80*
		R	8	231.93 ± 34.34	551.62 ± 82.17	276.94 ± 42.65
	Th1	W	6	249.82 ± 58.56	516.36 ± 40.77	255.52 ± 26.31
		C	3	244.30 ± 47.40	418.97 ± 64.24	264.37 ± 42.32
		S	7	251.28 ± 27.80	496.28 ± 44.88	372.93 ± 50.63*
		R	8	273.19 ± 61.56	460.77 ± 28.07	287.83 ± 41.26
	Viaat	W	9	255.52 ± 45.10	468.35 ± 144.79	241.69 ± 28.07
		C	9	256.36 ± 43.87	462.65 ± 130.45	253.06 ± 35.02
		S	7	273.17 ± 31.29	489.74 ± 38.37	392.88 ± 28.93*
		R	16	238.98 ± 44.85	530.35 ± 91.10	285.06 ± 38.56

Mean breath per minute (BPM) values are presented, +/– SD. *p < 0.05 effect of genotype for marked groups vs. all other groups in the same Cre-CKO/CR experiment, as determined by ANOVA and Student-Newman-Keuls post-hoc correction for multiple testing. Abbreviations, W, WT; C, CRE; F, FLOX; K, CKO; S, STOP; R, RESC. Additional parameters including Irregularity Score, Apnea Index, and uncompensated Tidal Volume were also quantified (Supplementary Table 2). Representative plethysmography traces are available (Supplementary Figure 2).

### Pharmacologic Manipulation of Excitatory, Inhibitory, and Modulatory Systems Affects the Breathing Rate Response to Hypoxia

Genetic manipulation of MeCP2 function within the excitatory, inhibitory, and modulatory populations demonstrated the critical requirement of MeCP2 function in these neuronal populations for normal HVD. To extend our understanding we pharmacologically manipulated these neurotransmitters to determine if the HVD could be improved within NULL mice. Previous work has shown that loss of MeCP2 function causes a cell-autonomous reduction of expression of the biosynthetic enzymes required for the synthesis of these specific neurotransmitters (25, 26, 33); therefore, we tested whether compounds that enhance signaling of these neurotransmitters (TH neurons: L-DOPA, carbidopa, desipramine; VIAAT neurons: tiagabine, baclofen, muscimol) would modulate the HVD. Additionally we tested ketamine, an NMDA receptor agonist, which has been reported to correct hyperexcitability within forebrain regions of mice lacking MeCP2 (34).

**Figure 3:**
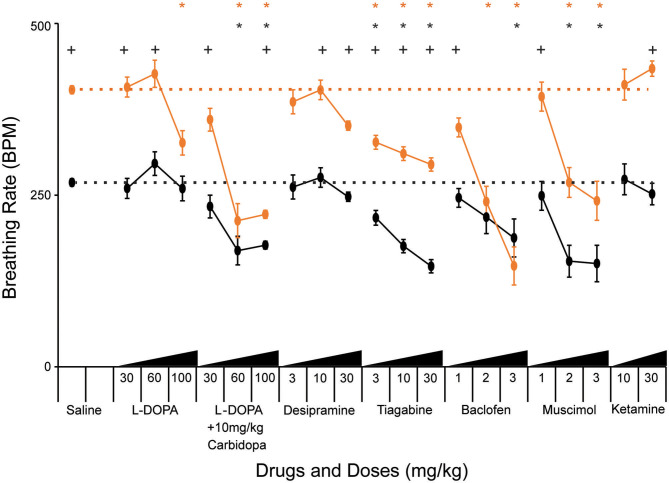
Pharmacological treatments modify breathing rate during acute hypoxia. “Declined breathing rate” (between 10 and 15 min after the onset of hypoxia) data is presented for 129B6F1 mice treated with saline and various concentrations of the indicated drugs 1 h before exposure to hypoxia. Values indicate mean +/– S.E.M. *p < 0.05 effect of drug vs. saline on breathing rate within genotype (black-WT, orange-NULL). +*p* < 0.05 effect of genotype within the indicated drug and dose. *p*-values determined by ANOVA with Bonferroni *post-hoc* correction for 61 multiple comparisons (40 for effect of drug vs. saline and 21 for effect of genotype at each drug and dose). Number of animals in each group is presented in Table 3.

Treatment with L-DOPA bypasses the biosynthetic deficit in the production of dopamine and norepinephrine that is present in NULL mice due to decreased Tyrosine Hydroxylase expression (26). Efficacy of L-DOPA treatment can be improved by co-administration with carbidopa, a structural analog of L-DOPA that inhibits DOPA decarboxylase present in the gut, allowing more L-DOPA in peripheral circulation to cross the blood brain barrier where it can improve noradrenergic and dopaminergic neuron function. Low doses of L-DOPA (30 and 60 mg/kg) had little impact on HVD. However, increased levels of L-DOPA (100 mg/kg) decreased the breathing rate following several minutes of hypoxic exposure (Figure 3, Table 3). Carbidopa improved the response and reduced the dosage of L-DOPA required to modify the breathing rate during hypoxia; however, treatment with high doses of L-DOPA with carbidopa also diminished the peak breathing rate during hypoxia (Figure 3, Table 3).

**Table 3:** Baseline and hypoxic breathing parameters following acute drug treatment.

**Drug**	**Dose mg/kg**	**Genotype**	***N***	**Baseline BPM**	**10% O2 Peak BPM**	**10% O2 Declined BPM**
Saline		WT	73	224.05 ± 32.61	495.13 ± 66.98	268.31 ± 47.62
		NULL	78	268.11 ± 36.67+	488.02 ± 39.95	403.26 ± 46.53+
L-DOPA	30	WT	8	200.73 ± 9.13	440.01 ± 82.86	259.72 ± 29.84
	60	WT	6	243.66 ± 33.19	514.42 ± 56.40	295.82 ± 70.39
	100	WT	8	227.36 ± 10.04	411.19 ± 71.78*	259.53 ± 35.82
	30	NULL	8	260.29 ± 21.86+	478.68 ± 56.41	407.29 ± 50.06+
	60	NULL	8	296.08 ± 36.12	501.84 ± 20.95	426.62 ± 22.63+
	100	NULL	8	266.57 ± 45.23	427.66 ± 55.62	326.09 ± 61.47*
L-DOPA + 10mg/kg Carbidopa	30	WT	12	251.99 ± 38.75	444.94 ± 71.41	233.39 ± 57.62
	60	WT	5	205.61 ± 21.63	338.77 ± 88.41*	169.07 ± 37.00*
	100	WT	34	204.61 ± 28.59*	324.14 ± 48.14*	177.01 ± 26.05*
	30	NULL	12	334.14 ± 22.04*+	492.57 ± 35.02	359.78 ± 57.40+
	60	NULL	7	244.11 ± 100.76	340.40 ± 111.44*	212.76 ± 64.47*
	100	NULL	36	219.16 ± 44.74*	312.25 ± 48.87*	221.92 ± 39.24*+
Desipramine	3	WT	4	199.18 ± 23.82	507.59 ± 70.93	261.73 ± 33.77
	10	WT	4	206.82 ± 14.09	529.00 ± 72.21	275.64 ± 29.45
	30	WT	6	219.48 ± 12.72	420.68 ± 35.47	247.50 ± 14.73
	3	NULL	4	266.09 ± 58.38	469.60 ± 22.87	385.83 ± 36.08
	10	NULL	4	289.39 ± 33.26	516.58 ± 33.67	403.16 ± 27.52+
	30	NULL	6	326.75 ± 67.87*	470.95 ± 22.90	351.02 ± 17.90+
Tiagabine	3	WT	22	194.53 ± 12.25*	430.29 ± 55.97*	216.92 ± 48.17*
	10	WT	15	189.07 ± 18.67*	376.44 ± 51.35*	175.68 ± 19.50*
	30	WT	4	256.41 ± 16.18	357.73 ± 16.94*	146.38 ± 21.06*
	3	NULL	20	289.96 ± 28.97+	460.32 ± 30.31	326.91 ± 47.03*+
	10	NULL	16	282.39 ± 30.76+	472.42 ± 56.26+	310.40 ± 49.91*+
	30	NULL	4	250.37 ± 23.00	465.69 ± 18.79+	294.49 ± 17.80*+
Baclofen	1	WT	8	200.67 ± 16.42	537.31 ± 39.75	245.95 ± 39.75
	2	WT	7	195.51 ± 11.63	517.88 ± 34.51	218.01 ± 39.98
	3	WT	4	204.46 ± 4.26	396.02 ± 86.35	187.62 ± 54.11*
	1	NULL	8	209.96 ± 26.95*	446.94 ± 29.74+	348.51 ± 37.86+
	2	NULL	6	237.42 ± 44.36	350.07 ± 78.26*+	240.36 ± 75.99*
	3	NULL	4	143.58 ± 35.03*	197.42 ± 14.49*	146.77 ± 56.90*
Muscimol	1	WT	8	217.09 ± 9.16	495.59 ± 73.38	248.87 ± 34.29
	2	WT	8	171.61 ± 30.81*	387.34 ± 86.31*	153.70 ± 66.62*
	3	WT	7	148.70 ± 49.41*	396.39 ± 71.50*	150.34 ± 32.11*
	1	NULL	8	277.90 ± 54.56	507.23 ± 87.24	393.38 ± 76.74+
	2	NULL	7	234.31 ± 20.17+	365.48 ± 68.62*	268.45 ± 54.91*
	3	NULL	8	188.31 ± 36.68*	370.08 ± 38.41*	241.65 ± 97.85*
Ketamine	10	WT	4	241.09 ± 15.72	501.78 ± 53.76	272.85 ± 22.41
	30	WT	4	223.04 ± 15.88	510.12 ± 83.80	251.58 ± 25.55
	10	NULL	4	282.47 ± 40.02	482.83 ± 53.74	410.80 ± 59.30
	30	NULL	2	292.04 ± 14.25	513.16 ± 18.61	434.15 ± 5.37+

Mean breath per minute (BPM) values are presented, +/– SD. *p < 0.05 effect of drug versus saline. +p < 0.05 effect of genotype within the same drug and dosage as determined by ANOVA with Bonferroni post-hoc correction for 61 multiple comparisons (40 for effect of drug vs. saline and 21 for effect of genotype at each drug and dose). Additional parameters including Irregularity Score, Apnea Index, and uncompensated Tidal Volume were also quantified (Supplementary Table 3).

Desipramine, a tricyclic antidepressant that inhibits reuptake of norepinephrine, has previously been tested in NULL mice and improves the incidence of apnea caused by loss of MeCP2 (13, 35). Treatment with desipramine (30 mg/kg) significantly improved HVD of NULL mice, with minimal impact on their peak breathing rate during hypoxia (Figure 3, Table 3).

Treatment of mice with tiagabine, a GABA reuptake inhibitor, improved HVD in NULL animals to levels closer to those of saline treated WT mice (Figure 3, Table 3). Similarly, the GABA_A_ and GABA_B_ receptor agonists muscimol and baclofen also improved the HVD of NULL animals. However, the dosages of muscimol and baclofen that showed significant reduction in breathing rate during exposure to hypoxia also caused significant decreases in the peak breathing rate during hypoxia. Dosages of tiagabine that improved HVD had less impact than muscimol or baclofen on the peak breathing rate during hypoxia (Figure 3, Table 3).

Both dosages of Ketamine that were tested showed no improvement in HVD, the higher 30 mg/kg dose trended toward a worsening of the impaired HVD (*p* = 0.01 ANOVA for effect of drug on NULL mice, not corrected for multiple comparisons) (Figure 3, Table 3).

While some treatments, such as L-DOPA without carbidopa, desipramine, baclofen, and ketamine, had little to no impact on the hypoxic breathing response in WT animals, several others such as L-DOPA with carbidopa, tiagabine, and muscimol also modified the WT hypoxic breathing response.

### Mice Lacking MeCP2 Function Have Decreased Hypoxia Induced Neuronal Activity

**Figure 4:**
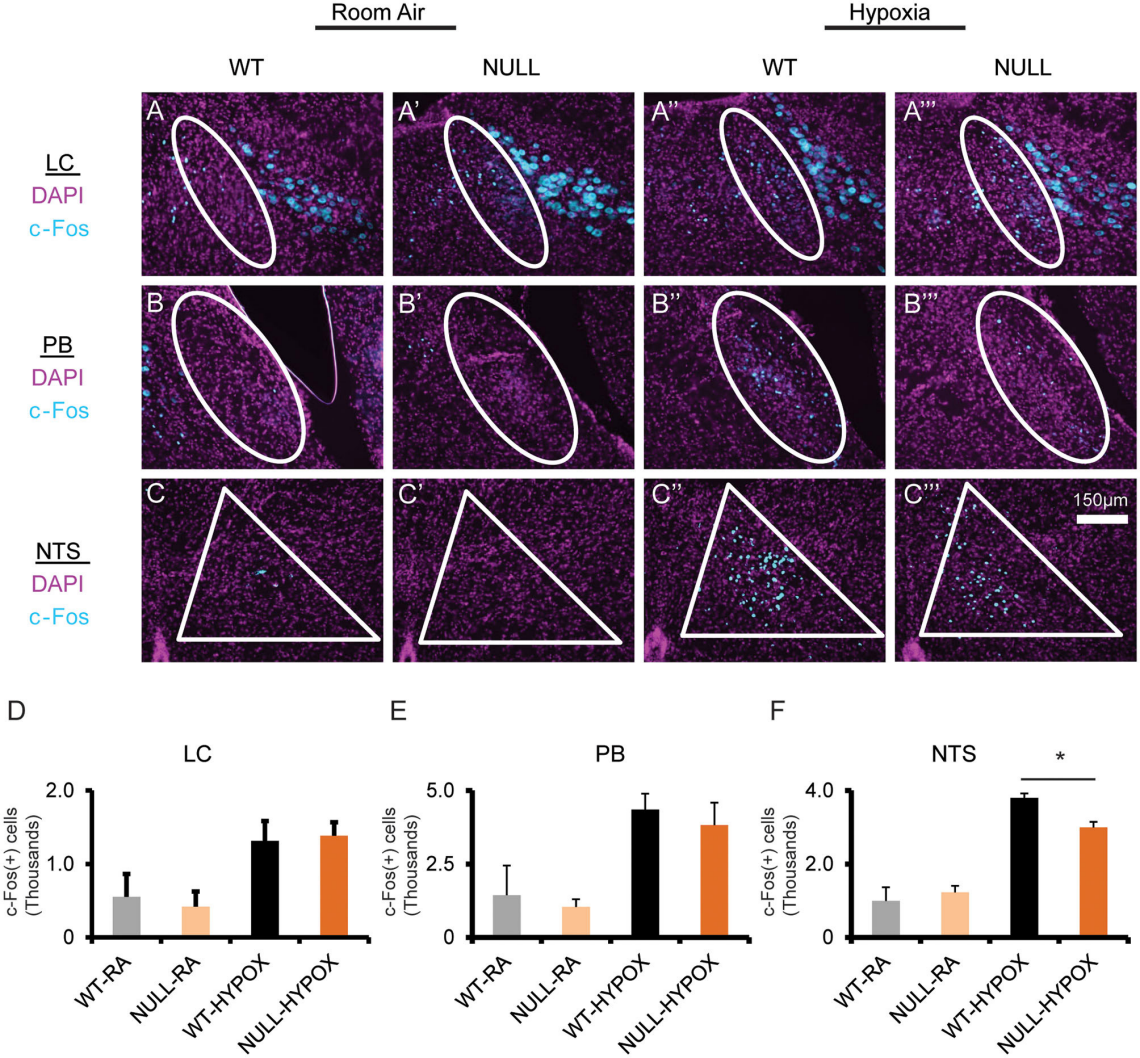
Mice lacking MeCP2 function exhibit decreased hypoxia induced neuronal activation. Treatment with hypoxia (10% O2, balance N2) for 3 h induces neuronal activation across several brainstem regions, as determined by immunofluorescent staining for c-Fos expression in coronal sections through the brainstem containing the locus coeruleus (LC) **(A–A”')**, Parabrachial Complex (PB) **(B–B”')**, and nucleus solitary tract (NTS) **(C–C”')**. Stereological quantification of c-Fos expressing nuclei identified an increase in neuronal activation in both WT and NULL mice during treatment with hypoxia in the LC **(D)**, PB **(E)**, and NTS **(F)**. However, the quantity of neurons activated in the NTS of NULL mice was 20% lower than WT littermates (Room Air treated WT *N* = 2, Room Air treated NULL *N* = 2, Hypoxia treated WT *N* = 3, Hypoxia treated NULL *N* = 3). Indicated brain regions outlined in white. *p < 0.05 for indicated comparison by One-Way ANOVA with Student-Newman-Keuls *post-hoc* correction for multiple comparisons. RA, room air; HYPOX, hypoxia. Bar graphs indicate mean values, error bars indicate SEM.

Finally, we wanted to determine if loss of MeCP2 modified hypoxia induced neuronal activation as an explanation of the abnormal HVD. Hypoxia induced neuronal activation was assayed by immunostaining for c-Fos reactivity in mice that were treated with hypoxic gas compared to mice allowed by continue breathing room air. We performed stereological quantification of c-Fos immunoreactivity in regions that are activated by exposure to hypoxia including the LC, PB, and NTS. All three anatomic regions showed an increase in c-Fos positive nuclei in both WT and in NULL animals; however, NULL mice had a 20% deficit in hypoxia induced c-Fos expressing nuclei in the NTS (Figure 4).

## Discussion

MeCP2 is required for an appropriate breathing rate response to acute hypoxic challenge. Genetic knockout and rescue of *Mecp2* within glutamatergic, GABAergic, and dopaminergic/noradrenergic neurons indicates that MeCP2 function is required in a distributed set of neurons for a normal breathing response to hypoxia. Genetic knockout and rescue of *Mecp2* within the caudal pons and medulla (using *HoxB1*-Cre) indicated that the critical neural circuitry for the normal HVD is within these brain regions (14, 22). In combination, this indicates that a neural circuit within the caudal pons and medulla comprised of excitatory, inhibitory, and dopaminergic/noradrenergic neurons underlie the HVD. Because within this region there are no dopaminergic neurons, it is likely that the MeCP2 requirement for normal HVD in TH-expressing neurons is solely within the noradrenergic neurons.

Other studies identified an increase in resting activity, as measured by c-Fos staining, within the NTS of NULL mice relative to WT controls; our current experiment is underpowered to confirm this in the room air condition (34). However, upon exposure to hypoxia, there is a clear increase in c-Fos expression in both genotypes, consistent with studies looking at brain regions activated by exposure to hypoxia (28). Interestingly, NULL mice have 20% fewer c-FOS expressing neurons within the NTS compared to WT controls. The c-FOS activation within the LC and PB did not show an effect size sufficient to be detected; however, the limited power of the present study prevents a definitive conclusion that these regions are not impacted in NULL mice. The impairment in hypoxia induced neuronal activation within the dorsal medulla's NTS, a key relay center in the processing of hypoxic stimuli, thus becomes an attractive candidate for contributions to the MeCP2 related impairment in HVD observed in NULL animals. The role for the NTS is also supported by previous anatomically focused conditional genetic studies which suggest key centers mediating the aberrant MeCP2 dependent hypoxic breathing phenotype do not extend beyond the rostral boundary of developmental expression of HoxB1 or HoxA4 (14, 22).

Excitatory/inhibitory balance within the NTS contributes to HVD (36). Inhibition of NMDA receptors within the NTS is thought to play a part in HVD, particularly via PDGF-beta signaling. This is due to observation of impaired HVD in mice heterozygous for a mutation in the PDGF-beta receptor as well as in mice lacking PDGF-beta expression in the brain (37, 38). Other studies have been able to demonstrate that treatment with the NMDA receptor agonist ketamine can ameliorate several phenotypes present in mice lacking MeCP2 including disrupted breathing and apnea (34, 39). We were unable to replicate the breathing improvement from ketamine treatment, potentially due to the limited number of animals tested, as well as to differences in dose and acute treatment vs. chronic dosing used in prior studies (Supplementary Table 3) (39). Similarly, we did not see any trend toward improvement of HVD from ketamine treatment.

Glutamatergic release in the NTS stimulated by hypoxia provides a pool of glutamate available for conversion to GABA and use for inhibitory signaling. The requirement of MeCP2 for GABA biosynthesis and GABAergic neuronal function suggest this as a likely contributor to the abnormal HVD observed in *Mecp2* mutant mice (25). Consistent with this hypothesis, modulation of GABAergic function within the NTS of rats by microinjection with GABA agonists and antagonists modulates the ventilatory response to hypoxia (36).

The tyrosine hydroxylase neurons within the central nervous system also are involved in HVD, as blocking dopamine activity with haloperidol prevents HVD from occurring (40). Furthermore, A2/C2 adrenergic neurons within the NTS begin to lose expression of TH in the absence of MeCP2 by 1 month of age, correlating with the onset of the abnormal HVD observed in *Mecp2* mutant mice (33). Optogenetic stimulation of C2 TH neurons genetically deficient for *VGLUT2* suggest that the TH and VGLUT2 populations overlap and that TH dependent modulation of breathing is dependent on VGLUT2 expression (41).

The neuronal circuits that control the breathing response to hypoxia span multiple anatomic regions, and categories of neurons. The initially normal HVD observed in young NULL mice implies that MeCP2 function is not essential to the establishment of these circuits, but that it is critical across multiple components for their maintenance as the deficit manifests by 4 weeks of age. Pharmacological treatment of NULL mice targeting excitatory, inhibitory, and modulatory neurons suggests that the impairment in HVD is due to decreased activity across each of the populations.

While not the main objective of the current study, we were also able to monitor additional breathing parameters of baseline breathing including apnea and irregularity of breathing (Supplementary Tables 1–3). Overall, we did observe an expected increase in the incidence of apnea in NULL mice relative to WT animals as well as effects and trends indicating increased breathing irregularity scores in NULL mice. These non HVD outcome measures presented with large variation making definitive conclusions from the genetic and pharmacological studies underpowered with respect to apnea and breathing irregularity. However, we did note that some of the pharmacological treatments, such as L-DOPA with carbidopa, baclofen, and muscimol, modified breathing under sustained hypoxia and resulted in worsening of apnea as well as irregularity. Interestingly, these treatments also blunted the peak breathing response to hypoxia while tiagabine and desipramine reduced the breathing rate during sustained hypoxia while sparing the peak breathing response to hypoxia and did not appear to aggravate the incidence of apnea or breathing irregularity.

These experiments attempted to dissect the neuronal substrates modified by the absence of MeCP2 and result in impaired HVD. In this sense, the impaired HVD can be considered another biomarker used as an indicator for neurological impairment due to the loss of MeCP2. Our findings are consistent with the pleiotropic action of MeCP2, and with the distributed nature of the brainstem circuitry that regulates HVD (22), and provide insight into the neuronal cell and circuit basis of breathing abnormalities in RTT. As these breathing abnormalities may underlie the sudden and unexpected death seen in RTT, dissecting the pathophysiology and developing targeted therapeutic interventions may be able to help prevent these deaths.

It is important to note that while the experiments looking to dissect the HVD impairment were largely performed using male mice, we did observe the phenotype in female HET animals. Among the key differences between male and female animals with *Mecp2* mutations is the mosaicism of MeCP2 expression within the neuronal populations under study due to *Mecp2* being X-linked and subject to X-chromosome inactivation. Other studies have contrasted the effects of genetic studies between the male and female models. Typically, the phenotypes observed in the female models are less severe due to their mosaicism, but differential benefit may be observed depending on the system that is targeted for genetic restoration (24, 27). Specifically, this contrast is apparent within the genetic rescue of MeCP2 within the excitatory and inhibitory neuronal populations performed by Meng et al. and Ure et. al.; female mice showed greater benefit from rescue in the excitatory neurons while male mice showed greater benefit from rescue in the inhibitory neurons (24, 27). This effect is likely due to activity imbalances within their respective networks upon restoration of MeCP2 with network components expressing varying degrees of mosaicism. Overall, these and previous findings are encouraging regarding development of potential therapies for RTT because they provide additional evidence of neuronal circuits that remain present but are poorly tuned in the absence of MeCP2. Pharmacological or genetic therapies have the potential to re-adjust these circuits to a more normal state; however, full rescue will likely require broad acting interventions that target multiple neuronal populations to achieve balanced activity.

## Data Availability Statement

The raw data supporting the conclusions of this article will be made available by the authors, without undue reservation.

## Ethics Statement

The animal study was reviewed and approved by Baylor College of Medicine Institutional Animal Care and Use Committee.

## Author Contributions

CW, T-WH, and JN contributed to conception and design of the study. CW, T-WH, JH, DP, and EA performed experiments and collected data. CW and CM developed analysis tools, CW analyzed the data. CM, RS, AI-I, XM, KU, and HZ, contributed to the genetic studies of *Vglut2, Viaat, and Th* conditional knock-out and rescue. CW and JN wrote the first draft of the manuscript. All authors contributed to manuscript revision, read, and approved the submitted version.

## Conflict of Interest

The authors declare that the research was conducted in the absence of any commercial or financial relationships that could be construed as a potential conflict of interest.
