# Supplementary material for: Loss of MeCP2 Function Across Several Neuronal Populations Impairs Breathing Response to Acute Hypoxia
Source: Front Neurol. 2020 Oct 30;11:593554. doi: 10.3389/fneur.2020.593554 (PMC7662121; doi:10.3389/fneur.2020.593554)
Supplement: Supplementary file 1 [file Data_Sheet_1.docx]

Supplementary Material

# Supplemental Tables

Table S1: Additional baseline breathing parameters in *Mecp2* mutant mice grouped by age, strain, and sex

Irregularity Score, Apnea Index (apneas per 10000 breaths) and uncompensated Tidal Volume values are presented, +/- SD. *p<0.05 effect of genotype (within each age, strain, and sex group). +p<0.05 effect of age (Male 129B6F1 only). ^p<0.05 effect of age*genotype (Male 129B6F1 only) as determined by ANOVA.

| Strain | Sex | Age | Genotype | N | Irregularity Score+ | | | | Apnea+^ | | | | Tidal Volume (uncomp) | | | |
| --- | --- | --- | --- | --- | --- | --- | --- | --- | --- | --- | --- | --- | --- | --- | --- | --- |
| 129B6F1 | Male | 3 wk | WT | 13 | 0.38 | ± | 0.59 |  | 167 | ± | 247 |  | 9.5 | ± | 15.3 |  |
|  |  |  | NULL | 12 | 0.70 | ± | 0.28 |  | 486 | ± | 176 | * | 4.5 | ± | 0.6 |  |
|  |  | 4 wk | WT | 8 | 0.11 | ± | 0.02 |  | 42 | ± | 30 |  | 6.9 | ± | 1.6 |  |
|  |  |  | NULL | 8 | 0.16 | ± | 0.06 | * | 118 | ± | 84 | * | 5.7 | ± | 0.6 |  |
|  |  | 5 wk | WT | 7 | 0.11 | ± | 0.03 |  | 16 | ± | 12 |  | 7.4 | ± | 0.7 |  |
|  |  |  | NULL | 6 | 0.16 | ± | 0.06 | * | 45 | ± | 16 | * | 8.5 | ± | 1.0 | * |
| 129B6F1 | Female | 2 yr | WT | 7 | 0.26 | ± | 0.06 |  | 20 | ± | 12 |  | 17.5 | ± | 1.4 |  |
|  |  |  | HET | 8 | 0.12 | ± | 0.04 | * | 12 | ± | 10 |  | 17.4 | ± | 1.8 |  |
| C57BL/6J | Male | 2 mo | WT | 10 | 0.23 | ± | 0.07 |  | 26 | ± | 33 |  | 8.5 | ± | 1.2 |  |
|  |  |  | NULL | 11 | 0.26 | ± | 0.09 |  | 112 | ± | 107 | * | 8.8 | ± | 1.6 |  |

Table S2: Additional baseline and breathing parameters from *Mecp2* conditional knockout and conditional rescue mice

Irregularity Score, Apnea Index (apneas per 10000 breaths) and uncompensated Tidal Volume values are presented, +/- SD. *p<0.05 effect of genotype for marked groups vs all other groups in the same Cre-CKO/CR experiment, as determined by ANOVA and Student-Newman-Keuls post-hoc correction for multiple testing. Abbreviations, W : WT, C : CRE, F : FLOX, K : CKO, S : STOP, R : RESC.

| CKO/CR | Cre | Genotype | N | Irregularity Score | | | | Apnea | | | | Tidal Volume (uncomp) | | | |
| --- | --- | --- | --- | --- | --- | --- | --- | --- | --- | --- | --- | --- | --- | --- | --- |
| CKO | Nestin | W | 11 | 0.22 | ± | 0.11 |  | 33 | ± | 59 |  | 8.7 | ± | 1.0 |  |
|  |  | C | 9 | 0.24 | ± | 0.10 |  | 45 | ± | 82 |  | 8.5 | ± | 1.5 |  |
|  |  | F | 11 | 0.20 | ± | 0.09 |  | 24 | ± | 36 |  | 9.6 | ± | 1.9 |  |
|  |  | K | 13 | 0.24 | ± | 0.12 |  | 68 | ± | 84 |  | 8.2 | ± | 1.8 |  |
|  | Vglut2 | W | 13 | 0.18 | ± | 0.03 |  | 5 | ± | 7 |  | 11.7 | ± | 1.7 |  |
|  |  | C | 15 | 0.18 | ± | 0.05 |  | 7 | ± | 9 |  | 11.6 | ± | 1.1 |  |
|  |  | F | 14 | 0.24 | ± | 0.07 |  | 18 | ± | 16 |  | 11.0 | ± | 1.3 |  |
|  |  | K | 11 | 0.19 | ± | 0.08 |  | 23 | ± | 17 |  | 15.0 | ± | 4.3 | * |
|  | Th1 | W | 8 | 0.17 | ± | 0.03 |  | 17 | ± | 16 |  | 10.1 | ± | 1.8 |  |
|  |  | C | 7 | 0.15 | ± | 0.04 |  | 4 | ± | 7 |  | 11.1 | ± | 2.7 |  |
|  |  | F | 8 | 0.21 | ± | 0.06 |  | 67 | ± | 54 | * | 8.3 | ± | 1.4 |  |
|  |  | K | 11 | 0.17 | ± | 0.04 |  | 19 | ± | 18 |  | 11.3 | ± | 2.2 |  |
|  | Viaat | W | 8 | 0.19 | ± | 0.05 |  | 10 | ± | 7 |  | 8.7 | ± | 0.7 |  |
|  |  | C | 5 | 0.16 | ± | 0.05 |  | 4 | ± | 6 |  | 9.3 | ± | 1.0 |  |
|  |  | F | 7 | 0.25 | ± | 0.09 |  | 15 | ± | 17 |  | 8.8 | ± | 0.9 |  |
|  |  | K | 6 | 0.21 | ± | 0.07 |  | 19 | ± | 9 |  | 10.4 | ± | 2.2 |  |
| CR | Vglut2 | W | 6 | 0.14 | ± | 0.03 |  | 23 | ± | 22 |  | 9.1 | ± | 1.8 |  |
|  |  | C | 9 | 0.19 | ± | 0.06 |  | 17 | ± | 19 |  | 8.4 | ± | 1.2 |  |
|  |  | N | 5 | 0.21 | ± | 0.10 |  | 62 | ± | 74 |  | 10.7 | ± | 2.7 |  |
|  |  | R | 8 | 0.27 | ± | 0.07 |  | 52 | ± | 26 |  | 8.0 | ± | 1.7 |  |
|  | Th1 | W | 6 | 0.16 | ± | 0.06 |  | 9 | ± | 5 |  | 8.6 | ± | 0.6 |  |
|  |  | C | 3 | 0.18 | ± | 0.08 |  | 29 | ± | 16 |  | 9.1 | ± | 1.5 |  |
|  |  | N | 7 | 0.20 | ± | 0.07 |  | 48 | ± | 39 |  | 10.8 | ± | 2.2 |  |
|  |  | R | 8 | 0.21 | ± | 0.07 |  | 59 | ± | 55 |  | 8.6 | ± | 1.0 |  |
|  | Viaat | W | 9 | 0.20 | ± | 0.09 |  | 10 | ± | 10 |  | 9.0 | ± | 1.5 |  |
|  |  | C | 9 | 0.16 | ± | 0.07 |  | 7 | ± | 9 |  | 8.8 | ± | 2.4 |  |
|  |  | N | 7 | 0.24 | ± | 0.06 |  | 55 | ± | 24 |  | 9.6 | ± | 2.2 |  |
|  |  | R | 16 | 0.18 | ± | 0.08 |  | 33 | ± | 34 |  | 8.9 | ± | 1.3 |  |

Table S3: Additional baseline breathing parameters following acute drug treatment

Irregularity Score, Apnea Index (apneas per 10000 breaths) and uncompensated Tidal Volume values are presented, +/- SD. *p<0.05 effect of drug versus saline. +p<0.05 effect of genotype within the same drug and dosage as determined by ANOVA with Bonferroni post-hoc correction for 61 multiple comparisons (40 for effect of drug versus saline and 21 for effect of genotype at each drug and dose)

| Drug | Dose mg/kg | Genotype | N | Irregularity Score | | | | Apnea | | | | Tidal Volume (uncomp) | | | |
| --- | --- | --- | --- | --- | --- | --- | --- | --- | --- | --- | --- | --- | --- | --- | --- |
| Saline |  | WT | 73 | 0.15 | ± | 0.06 |  | 22 | ± | 25 |  | 8.5 | ± | 1.5 |  |
|  |  | NULL | 78 | 0.17 | ± | 0.07 |  | 45 | ± | 33 | + | 10.1 | ± | 2.3 | + |
| L-DOPA | 30 | WT | 8 | 0.15 | ± | 0.03 |  | 31 | ± | 24 |  | 8.7 | ± | 0.8 |  |
|  | 60 | WT | 6 | 0.19 | ± | 0.04 |  | 18 | ± | 8 |  | 9.6 | ± | 1.2 |  |
|  | 100 | WT | 8 | 0.18 | ± | 0.02 |  | 22 | ± | 6 |  | 10.0 | ± | 1.1 |  |
|  | 30 | NULL | 8 | 0.18 | ± | 0.08 |  | 55 | ± | 28 |  | 10.6 | ± | 1.8 |  |
|  | 60 | NULL | 8 | 0.19 | ± | 0.10 |  | 90 | ± | 110 |  | 10.7 | ± | 2.3 |  |
|  | 100 | NULL | 8 | 0.20 | ± | 0.05 |  | 77 | ± | 67 |  | 11.6 | ± | 2.6 |  |
| L-DOPA + 10mg/kg Carbidopa | 30 | WT | 12 | 0.15 | ± | 0.04 |  | 29 | ± | 12 |  | 8.6 | ± | 1.4 |  |
|  | 60 | WT | 5 | 0.15 | ± | 0.04 |  | 56 | ± | 61 |  | 6.2 | ± | 0.7 | * |
|  | 100 | WT | 34 | 0.17 | ± | 0.06 |  | 80 | ± | 94 |  | 6.4 | ± | 1.1 | * |
|  | 30 | NULL | 12 | 0.21 | ± | 0.03 |  | 42 | ± | 24 |  | 9.5 | ± | 1.3 |  |
|  | 60 | NULL | 7 | 0.44 | ± | 0.48 |  | 253 | ± | 265 | * | 7.2 | ± | 2.3 | * |
|  | 100 | NULL | 36 | 0.31 | ± | 0.19 | + | 232 | ± | 165 | *+ | 7.8 | ± | 1.6 | *+ |
| Desipramine | 3 | WT | 4 | 0.18 | ± | 0.03 |  | 30 | ± | 27 |  | 9.1 | ± | 1.3 |  |
|  | 10 | WT | 4 | 0.14 | ± | 0.02 |  | 7 | ± | 7 |  | 9.8 | ± | 0.9 |  |
|  | 30 | WT | 6 | 0.14 | ± | 0.02 |  | 30 | ± | 33 |  | 9.8 | ± | 0.7 |  |
|  | 3 | NULL | 4 | 0.16 | ± | 0.03 |  | 87 | ± | 26 |  | 8.8 | ± | 1.1 |  |
|  | 10 | NULL | 4 | 0.17 | ± | 0.05 |  | 48 | ± | 34 |  | 10.4 | ± | 2.0 |  |
|  | 30 | NULL | 6 | 0.16 | ± | 0.04 |  | 32 | ± | 21 |  | 11.2 | ± | 1.5 |  |
| Tiagabine | 3 | WT | 22 | 0.16 | ± | 0.03 |  | 21 | ± | 11 |  | 9.2 | ± | 0.8 |  |
|  | 10 | WT | 15 | 0.16 | ± | 0.07 |  | 53 | ± | 99 |  | 8.2 | ± | 1.2 |  |
|  | 30 | WT | 4 | 0.19 | ± | 0.04 |  | 12 | ± | 13 |  | 5.8 | ± | 0.2 | * |
|  | 3 | NULL | 20 | 0.20 | ± | 0.07 |  | 34 | ± | 27 |  | 12.9 | ± | 1.0 | *+ |
|  | 10 | NULL | 16 | 0.14 | ± | 0.06 |  | 42 | ± | 47 |  | 10.8 | ± | 2.2 | + |
|  | 30 | NULL | 4 | 0.12 | ± | 0.01 |  | 62 | ± | 12 |  | 10.3 | ± | 0.7 | + |
| Baclofen | 1 | WT | 8 | 0.15 | ± | 0.06 |  | 23 | ± | 14 |  | 9.3 | ± | 1.5 |  |
|  | 2 | WT | 7 | 0.16 | ± | 0.04 |  | 18 | ± | 10 |  | 9.5 | ± | 1.1 |  |
|  | 3 | WT | 4 | 0.16 | ± | 0.03 |  | 12 | ± | 5 |  | 9.4 | ± | 1.5 |  |
|  | 1 | NULL | 8 | 0.14 | ± | 0.04 |  | 27 | ± | 13 |  | 11.3 | ± | 1.8 |  |
|  | 2 | NULL | 6 | 0.13 | ± | 0.05 |  | 54 | ± | 51 |  | 11.8 | ± | 2.4 |  |
|  | 3 | NULL | 4 | 0.70 | ± | 0.63 |  | 377 | ± | 286 | * | 8.8 | ± | 3.0 |  |
| Muscimol | 1 | WT | 8 | 0.20 | ± | 0.06 |  | 8 | ± | 5 |  | 8.4 | ± | 1.1 |  |
|  | 2 | WT | 8 | 1.75 | ± | 1.36 | * | 1124 | ± | 608 | * | 7.8 | ± | 1.8 |  |
|  | 3 | WT | 7 | 2.91 | ± | 2.33 | * | 1249 | ± | 577 | * | 6.4 | ± | 1.6 | * |
|  | 1 | NULL | 8 | 0.24 | ± | 0.18 |  | 100 | ± | 215 |  | 10.9 | ± | 2.1 |  |
|  | 2 | NULL | 7 | 0.43 | ± | 0.30 |  | 384 | ± | 209 | * | 7.1 | ± | 1.4 | * |
|  | 3 | NULL | 8 | 2.61 | ± | 2.88 | * | 1031 | ± | 531 | * | 5.5 | ± | 0.7 | * |
| Ketamine | 10 | WT | 4 | 0.22 | ± | 0.04 |  | 11 | ± | 5 |  | 10.2 | ± | 0.8 |  |
|  | 30 | WT | 4 | 0.21 | ± | 0.04 |  | 22 | ± | 27 |  | 11.3 | ± | 0.4 | * |
|  | 10 | NULL | 4 | 0.24 | ± | 0.08 |  | 103 | ± | 88 |  | 11.3 | ± | 2.3 |  |
|  | 30 | NULL | 2 | 0.24 | ± | 0.18 |  | 338 | ± | 434 |  | 18.0 | ± | 1.6 | * |

# Supplemental Figures


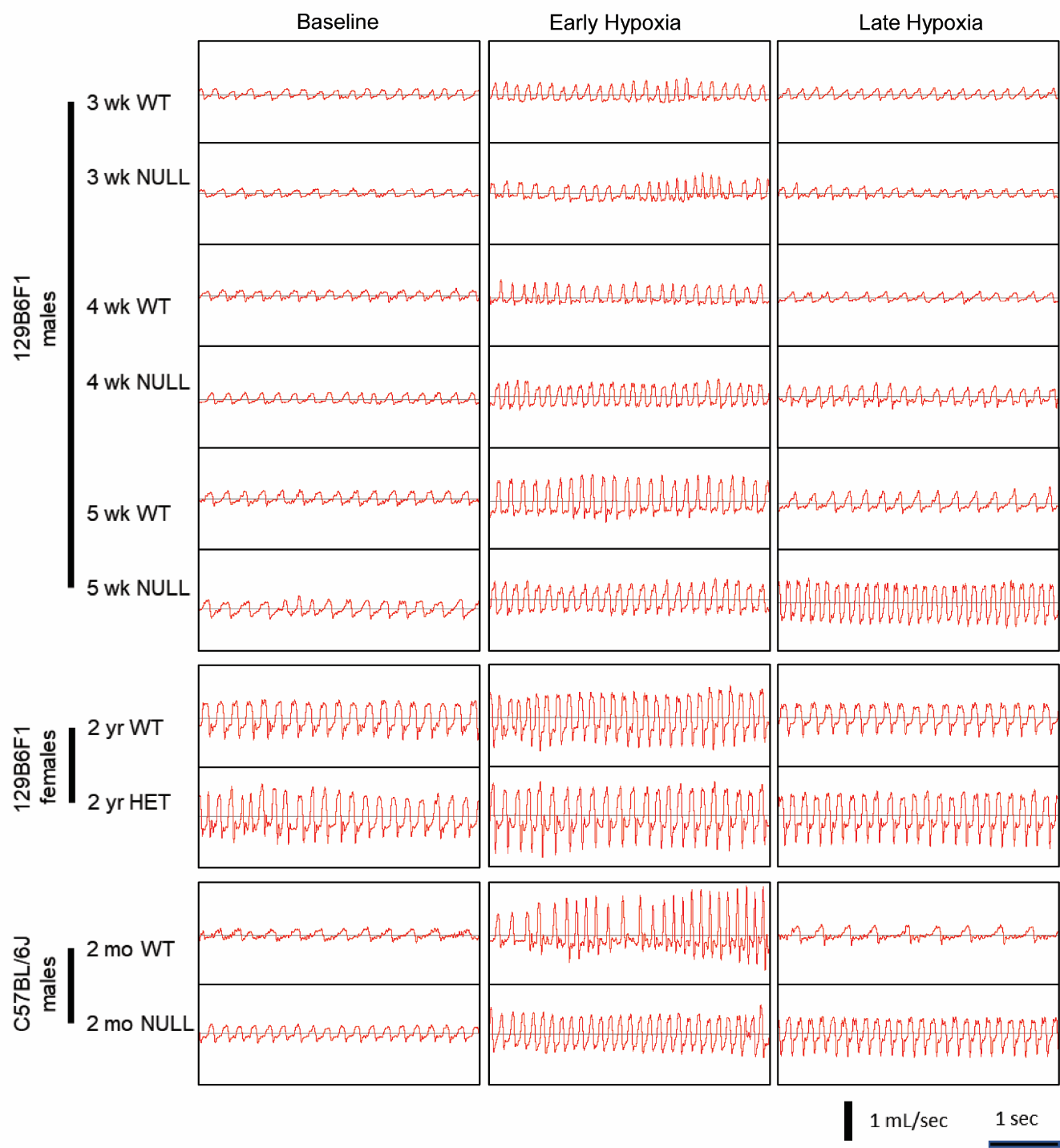


Figure S1: Representative plethysmograph traces showing breathing during baseline and hypoxic conditions for *Mecp2* mutant mice across age, strain, and sex

Red lines illustrate the box-flow of air into and out of the chamber from which breathing parameters can be derived. “Baseline” is breathing under normoxic conditions prior to exposure to 10% O_2_, “Early Hypoxia” is breathing within the first five minutes of exposure to 10% O_2_ when peak breathing rates are typically observed, “Late Hypoxia is breathing during minutes 10-15 of exposure to 10% O_2_ when breathing rates have declined in wildtype animals. Traces are representative for the indicated groups with summary data presented in Figure 1 and Table 1.


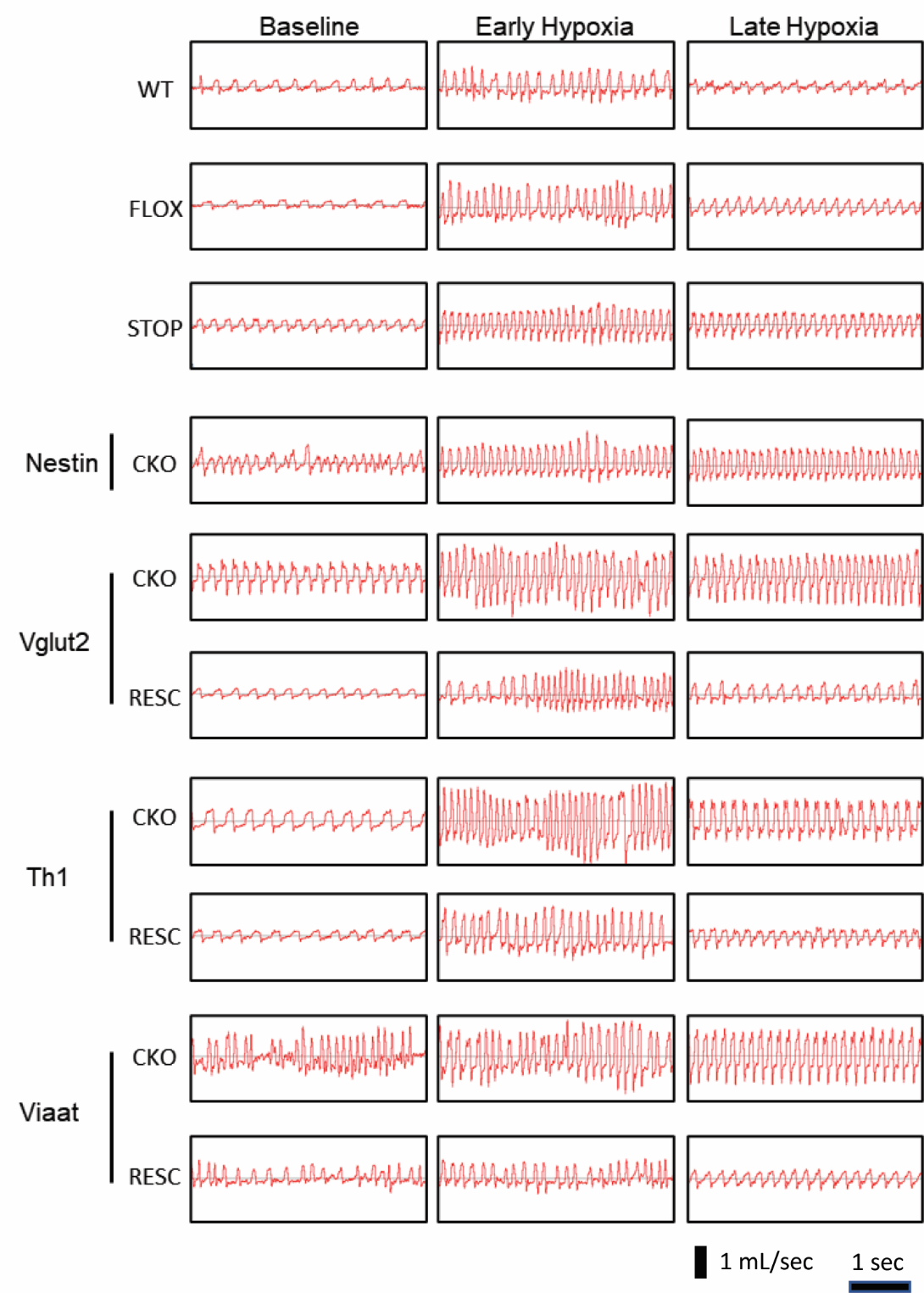


Figure S2: Representative plethysmograph traces showing breathing during baseline and hypoxic conditions for *Mecp2* CKO and CR mice

Red lines illustrate the box-flow of air into and out of the chamber from which breathing parameters can be derived. “Baseline” is breathing under normoxic conditions prior to exposure to 10% O_2_, “Early Hypoxia” is breathing within the first five minutes of exposure to 10% O_2_ when peak breathing rates are typically observed, “Late Hypoxia is breathing during minutes 10-15 of exposure to 10% O_2_ when breathing rates have declined in wildtype animals. Traces are representative for the indicated groups with summary data presented in Figure 1 (Nestin) Figure 2 (Vglut2, Th1, Viaat) and Table 2.
